# Case Report: Primary Hypothyroidism Associated With Lutetium 177-DOTATATE Therapy for Metastatic Paraganglioma

**DOI:** 10.3389/fendo.2020.587065

**Published:** 2021-01-21

**Authors:** Sriram Gubbi, Mohammad Al-Jundi, Jaydira Del Rivero, Abhishek Jha, Marianne Knue, Joy Zou, Baris Turkbey, Jorge Amilcar Carrasquillo, Emily Lin, Karel Pacak, Joanna Klubo-Gwiezdzinska, Frank I-Kai Lin

**Affiliations:** ^1^ Metabolic Diseases Branch, National Institute of Diabetes and Digestive and Kidney Diseases, National Institutes of Health, Bethesda, MD, United States; ^2^ Department of Endocrinology, Eunice Kennedy Shriver National Institute of Child and Human Development, National Institutes of Health, Bethesda, MD, United States; ^3^ Developmental Therapeutics Branch, National Cancer Institute, National Institutes of Health, Bethesda, MD, United States; ^4^ Molecular Imaging Program, National Cancer Institute, National Institutes of Health, Bethesda, MD, United States; ^5^ Department of Radiology, Memorial Sloan Kettering Cancer Center, New York, NY, United States; ^6^ Davis Senior High School, Davis, CA, United States

**Keywords:** DOTATATE, Lutathera, hypothyroidism, peptide receptor radionuclide therapy, paraganglioma

## Abstract

**Background:**

Lutetium 177 (^177^Lu) - DOTATATE is a form of peptide receptor radionuclide therapy (PRRT) utilized in the treatment of neuroendocrine tumors. Data on ^177^Lu-DOTATATE-induced thyroid dysfunction is limited.

**Case Description:**

A 29-year-old male with *SDHB* positive metastatic paraganglioma enrolled under the ^177^Lu-DOTATATE trial (NCT03206060) underwent thyroid function test (TFT) evaluation comprised of thyroid stimulating hormone (TSH) and free thyroxine (FT4) immunoassay measurements per protocol prior to ^177^Lu-DOTATATE therapy. The TSH was suppressed [<0.01 µIU/ml (0.27–4.2 µIU/ml)], and FT4 was normal [1.3 ng/dl (0.9–1.7 ng/dl)]. The TSH receptor antibody and thyroid stimulating immunoglobulin index were undetectable [<1 IU/L (≤1.75 IU/L), and <1 (≤1.3) respectively], while the anti-thyroid peroxidase (anti-TPO) and anti-thyroglobulin (anti-Tg) antibodies were elevated [605 IU/ml (0.0–34.9 IU/ml), and 178 IU/ml (0.0-40.0 IU/ml) respectively]. Mass spectrometry on a stored (-80°C) plasma sample obtained one-month pre-PRRT revealed elevated total triiodothyronine (TT3) [235 ng/dl (65–193 ng/dl)] and FT4 [3.9 ng/dl (1.2–2.9 ng/dl)] levels. The patient was diagnosed with Hashimoto’s thyrotoxicosis. However, the patient was asymptomatic. One month after the first dose of 200mCi ^177^Lu-DOTATATE, the patient noted fatigue and a 2.6 Kg weight gain. The TSH (73.04 µIU/ml), anti-TPO antibodies (>1,000 IU/ml), and anti-Tg antibodies (668 IU/ml) had substantially increased, with reductions in FT4 (0.3 ng/dl) and TT3 [54 ng/dl (87–169 ng/dl)]. Diagnostic gallium 68 - DOTATATE positron emission tomography-computed tomography performed prior to ^177^Lu-DOTATATE treatment revealed diffuse thyroid uptake. Post-therapy single-photon emission computed tomography also revealed diffuse uptake of ^177^Lu-DOTATATE in the thyroid gland. Levothyroxine therapy was initiated, and the patient’s symptoms resolved.

**Summary:**

We report, for the first time, a patient with asymptomatic primary hyperthyroidism who rapidly developed symptomatic primary hypothyroidism 1 month after ^177^Lu-DOTATATE therapy, accompanied by marked changes in TFTs and thyroid auto-antibody titers, with functional imaging evidence of diffuse uptake of ^177^Lu-DOTATATE in the thyroid gland.

**Conclusions:**

Thyroid dysfunction can be associated with PRRT. Thyroid uptake patterns on pre-treatment diagnostic somatostatin analog scans might predict individual susceptibility to PRRT-associated TFT disruption. Therefore, periodic evaluation of TFTs should be considered in patients receiving PRRT.

## Introduction

Peptide receptor radionuclide therapy (PRRT) is a form of targeted therapy that has demonstrated substantial efficacy in the treatment of neuroendocrine tumors (NETs) (1, 2). PRRT targets cells that possess high concentrations of somatostatin receptors (SSTRs), such as NETs, by utilizing a radiolabeled peptide (somatostatin) molecule (3). The somatostatin analog component of the molecule binds to the SSTRs and facilitates the delivery of the radionuclide directly to the tumor cells (2). Various analogs of PRRT possess theranostic properties, which is the ability to integrate diagnostic and therapeutic functions within the same pharmaceutical platform (4).

Lutetium 177 (^177^Lu) - DOTA-DPhe1, Tyr3-octreotate (DOTATATE) (Lutathera^®^, Advanced Accelerator Applications, Saint-Genis-Pouilly, France) is a form of PRRT with the highest affinity to SSTR-2. It has been successfully utilized in treating gastroenteropancreatic NETs and was recently shown to markedly prolong progression-free survival among patients with advanced, well-differentiated midgut NETs in the NETTER-1 phase 3 trial (2). Additionally, this agent has demonstrated substantial safety and efficacy based on data from 1200 patients treated for gastroenteropancreatic and bronchial NETs (5). ^177^Lu-DOTATATE has also been effectively utilized in the treatment of inoperable, metastatic pheochromocytomas and paragangliomas (PPGLs) (6, 7). ^177^Lu radionuclide exerts its anti-tumor effects by emitting medium energy beta particles and has a maximal tissue penetration of 2 mm (8). ^177^Lu-DOTATATE therapy is associated with several adverse effects, with nausea and vomiting being the most common adverse effects. Other adverse effects include fatigue/asthenia, abdominal pain, diarrhea, loss of appetite, musculoskeletal pain, headaches, flushing, dizziness, alopecia, cough, nephrotoxicity, hematotoxicity (leukopenia, thrombocytopenia, and lymphopenia), cardiotoxicity, hepatotoxicity, and acute hypertensive crisis (2, 7). ^177^Lu-DOTATATE therapy has also been associated with the disruption of endocrine function, although these effects are extremely rare and is often transient (9). We report, for the first time, a patient with primary hyperthyroidism who rapidly progressed to primary hypothyroidism after the first dose of ^177^Lu-DOTATATE therapy. A written informed consent was obtained from the patient for the publication of any potentially identifiable images or data included in this article.

## Case Description

A 29-year-old male with metastatic paraganglioma with succinate dehydrogenase subunit B (*SDHB*) germline pathogenic variant was enrolled in the ^177^Lu-DOTATATE trial (ClinicalTrials.gov identifier: NCT03206060) for the treatment of inoperable, metastatic PPGL at our center. As a part of the protocol, the patient underwent baseline thyroid function test (TFT) comprised of thyroid stimulating hormone (TSH) and free thyroxine (FT4) evaluation on the day of the first cycle of therapy, just prior to ^177^Lu-DOTATATE administration. The TSH level was suppressed [<0.01 µIU/ml (0.27–4.2 µIU/ml)], and the level was normal [1.3 ng/dl (0.9–1.7 ng/dl)] based on an immunoassay measurement. A repeat measurement of TSH and FT4 immunoassay revealed values of <0.01 µIU/ml and 1.2 ng/dl, respectively. TSH receptor antibody (TrAb) and thyroid stimulating immunoglobulin index were <1 IU/L (≤1.75 IU/L), and <1 (≤1.3), respectively. Anti-thyroid peroxidase (anti-TPO) and anti-thyroglobulin (anti-Tg) antibodies were 605 IU/ml (0.0–34.9 IU/ml), and 178 IU/ml (0.0-40.0 IU/ml), respectively. The patient complained of headaches, dizziness, and back pain, all of which were attributed to metastatic, biochemically active paragangliomas. The patient denied heat intolerance, weight loss, palpitations, sweating, diarrhea, changes in the appearance of his eyes, redness, eye pain or excessive tearing, neck pain, dysphagia, voice change, visual disturbances, skin changes, or diarrhea. Although multiple family members were affected with several non-thyroid malignancies, there was no family history of thyroid disorders or malignancies. The only form of radiation that the patient had received to the head and neck region was a computed tomography (CT) scan. His past medical history was relevant for SDHB-related metastatic paraganglioma (left glomus vagale, abdominal and hypopharyngeal lesions, and multiple bony metastases), Beckwith-Wiedemann syndrome (a genetic syndrome characterized by macrosomia and hemihypertrophy of the body, macroglossia, and increased risk for several embryonal tumors, and is usually caused due to cytogenetic abnormalities in chromosome 11p15) (10), and a chest neuroblastoma. The patient denied history of smoking, alcohol consumption, or drug use. Physical examination was unrevealing for lid lag, proptosis, conjunctival hyperemia or chemosis. The thyroid gland was of normal size without tenderness on palpation, and there was no cervical lymphadenopathy. The skin examination and deep tendon reflexes were normal. The heart rate was normal (70 beats per minute) with regular rate and rhythm. There were no tremors observed either in the tongue or in the upper extremities. There was slight enlargement of left hand and foot from BW syndrome. The patient was initially diagnosed with subclinical hyperthyroidism and was monitored without therapy. After endocrine consultation was requested for the patient, it was noted that the total triiodothyronine (T3) values were not measured. Therefore, FT4 and total T3 were measured using mass spectrometry along with TSH using immunoassay on a plasma sample stored at −80°C that was obtained 1 month prior to ^177^Lu-DOTATATE therapy. In this sample, the TSH was suppressed [<0.02 µIU/ml (0.27–4.2 µIU/ml)], FT4 was elevated [3.9 ng/dl (1.2–2.9 ng/dl)], along with an total T3 of 235 ng/dl (65–193 ng/dl), confirming a diagnosis of primary hyperthyroidism in retrospect, biochemically manifesting as thyrotoxic phase of Hashimoto’s thyroiditis.

**Figure 1:**
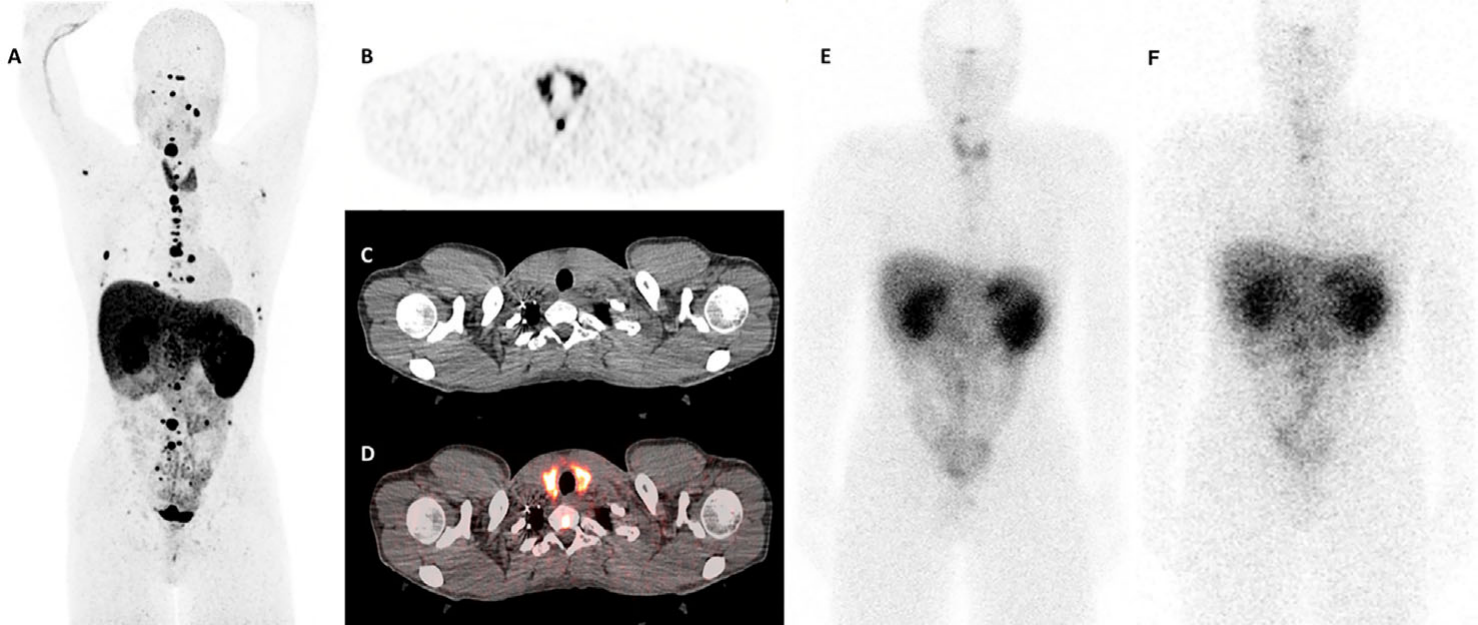
Thyroid uptake patterns in the patient before and after ^177^Lu-DOTATATE therapy. **(A)** A whole body (maximum intensity projection) view on the diagnostic ^68^Ga-DOTATATE scan demonstrating multiple paragangliomas as well as a diffuse uptake in the thyroid gland with a maximum standardized uptake value (SUVmax) of 14.3. **(B–D)** Axial slices at the thyroid level: **(B)** Positron emission tomography (PET) scan only. **(C)** Computed tomography (CT) scan only. **(D)** Fused PET and CT images. Images **(B, D)** demonstrate diffuse uptake in the thyroid gland. **(E, F)** The 24-h post-treatment whole body single-photon emission computed tomography (SPECT) scans demonstrating diffuse uptake of ^177^Lu-DOTATATE in the thyroid gland **(E)** After the first cycle of therapy, and **(F)** after the second cycle of therapy.

One month after the first cycle of 200mCi dose of ^177^Lu-DOTATATE therapy, the patient developed new onset fatigue and experienced a weight gain of 2.6 kg. Physical examination revealed normal skin, heart rate, and deep tendon reflexes. The thyroid examination was unremarkable with no cervical lymphadenopathy. At this point, the TSH had substantially increased (73.04 µIU/ml), along with reduction in the levels of FT4 (0.3 ng/dl) on immunoassay, and reduction in total T3 levels [54 ng/dl (87–169 ng/dl)] on mass spectrometry. Anti-TPO antibodies were >1,000 IU/ml, and anti-Tg antibodies were 668 IU/ml (**Table 1**). Subsequently, weight-based levothyroxine therapy was initiated. On follow-up visits, the TFTs normalized and his symptoms improved. A diagnostic gallium 68 (^68^Ga)-DOTATATE positron emission tomography-computed tomography (PET/CT) that was performed prior to the initiation of ^177^Lu-DOTATATE therapy revealed diffuse increase in the uptake in the entire thyroid gland, with a maximum standardized uptake value (SUVmax) of 14.3 (**Figures 1A–D**). A post-treatment single-photon emission computed tomography (SPECT) scans revealed diffuse uptake of ^177^Lu-DOTATATE in the thyroid gland (**Figures 1E, F**). Since then, the patient has continued his enrollment in the ^177^Lu-DOTATATE trial and is on replacement levothyroxine therapy with serial monitoring of TFTs.

**Table 1:** Thyroid function tests in the patient before and after ^177^Lu-DOTATATE therapy.

Time course	TSH (0.27–4.2 µIU/ml)	Free T4 (0.9–1.7 ng/dl)	Total T3* (65–193 ng/dl)	Anti-TPO Ab (0.0–34.9 IU/ml)	Anti-Tg Ab (0.0-40.0 IU/ml)	TrAb (≤1.75 IU/L)	TSI index (≤1.3)
Before ^177^Lu-DOTATATE therapy	<0.01 µIU/ml	1.3 ng/dl	235 ng/dl	605 IU/ml	178 IU/ml	<1 IU/L	<1
One month after the first dose of ^177^Lu-DOTATATE therapy	73.04 µIU/ml	0.3 ng/dl	54 ng/dl**	>1,000 IU/ml	668 IU/ml	Not measured	Not measured

*Total T3 measurements were performed using mass spectrometry. Free T4 and TSH values were measured using immunoassay.

**Total T3 at this time had a different reference range (87–169 ng/dl) due to a change in the mass spectrometry assays at our institution.

TSH, Thyroid stimulating hormone; T4, Thyroxine; T3, Triiodothyronine; TPO, Thyroid peroxidase; Tg, Thyroglobulin; TrAb, TSH receptor antibody; TSI, Thyroid stimulating immunoglobulin.

## Discussion

We report a patient with an initial diagnosis of asymptomatic primary hyperthyroidism who went on to develop overt, symptomatic primary hypothyroidism with marked changes in the TFTs and anti-thyroid antibody titers over a span of 1 month after the initiation of ^177^Lu-DOTATATE therapy, along with the imaging evidence of increased thyroid uptake of ^177^Lu-DOTATATE on SPECT imaging. Reports on sustained thyroid dysfunction following ^177^Lu-DOTATATE therapy are exceedingly rare and, to the best of our knowledge, a switch from hyperthyroidism to hypothyroidism associated with ^177^Lu-DOTATATE therapy, along with complementary functional imaging evidence has not been previously reported in the literature. However, it is not unlikely that the patient might have been on the natural course of Hashimoto’s thyroiditis from thyrotoxic phase toward hypothyroid phase irrespective of ^177^Lu-DOTATATE therapy, and the usual time course for such progression tends to be 1–24 months (11). However, the temporal association of marked changes in TFTs as well as a significant increase of anti-thyroid antibody titers associated with ^177^Lu-DOTATATE therapy, along with evidence of increased ^177^Lu-DOTATATE uptake on the SPECT imaging likely suggests a contribution of PRRT to thyroid disruption. Moreover, development of autoimmune thyroiditis has in fact been reported following ^177^Lu-DOTATATE therapy (9). In a Dutch cohort treated with ^177^Lu-DOTATATE for various forms of NETs, Teunissen et al. evaluated the pituitary-thyroid axis in 66 patients over a follow-up period of 12–24 months (9). The mean FT4 values changed from 1.38 ng/dl to 1.21 ng/dl over the treatment course, while there were no significant changes in TSH and total T3 levels. Two patients developed primary hypothyroidism: one patient developed anti-TPO antibody-positive hypothyroidism after the third cycle of treatment, while the other patient gradually developed hypothyroidism, needing hormone replacement 3.5 years after PRRT. However, patients with prior TFT abnormalities were excluded in this study.

The plausible mechanism for the rapid progression from hyperthyroid to hypothyroid phase in this patient could have been due to thyroid parenchymal destruction induced by ^177^Lu-DOTATATE therapy followed by accelerated autoimmune destruction. Prior to the treatment with ^177^Lu-DOTATATE, the patient had primary hyperthyroidism, and the presence of elevated anti-TPO and anti-Tg antibodies and an undetectable TrAb, suggested that the patient was likely in the thyrotoxicosis phase of Hashimoto’s (autoimmune) thyroiditis. ^177^Lu-DOTATATE therapy may have caused further damage to the thyroid follicles leading to increased exposure of thyroid parenchymal antigens (TPO, Tg), which in turn may have enhanced autoimmune-mediated destruction facilitating rapid progression toward overt hypothyroidism. This could explain the sudden increase in anti-TPO and anti-Tg antibodies, as well as TSH levels along with a concomitant reduction in FT4 and total T3 levels observed after ^177^Lu-DOTATATE therapy. Another differential diagnosis to consider in this patient would be antibody-negative Graves’ disease, which can be prevalent in 3%–5% of Graves’ disease patients, especially among those with new diagnosis or those with milder forms of the disease (12).

The exact mechanism of ^177^Lu-DOTATATE-associated thyroid disruption has not been elucidated. Cytotoxicity caused by the ^177^Lu radionuclide is possible, as somatostatin analogs have been previously shown to localize in the thyroid gland (13). In a retrospective analysis on a cohort of 237 patients who underwent ^68^Ga-DOTATATE imaging to localize unknown or metastatic NETs, 26 (11%) patients had an abnormal thyroid uptake, with 14 (54%) patients having focal uptake and 12 (46%) patients having diffuse thyroid uptake based on SUVmax measurements on the PET/CT (13). Among the patients with diffuse thyroid uptake, 42% had a history of hypothyroidism. Our patient was also found to have diffuse thyroid uptake on the diagnostic ^68^Ga-DOTATATE obtained prior to ^177^Lu-DOTATATE therapy, but he had hyperthyroidism at the time of diagnosis. ^68^Ga-DOTATOC, a somatostatin analog that predominantly targets SSTR-2 and SSTR-5 has demonstrated increased uptake in five of eight cases of Hashimoto’s thyroiditis (14). In the present case, although the thyroid uptake of ^68^Ga-DOTATATE was less than paraganglioma uptake on the baseline DOTATATE PET scan (**Figure 1A**), this finding was reversed in the whole body scintigraphy performed at 24-h post ^177^Lu-DOTATATE administration, with higher uptake in the thyroid gland compared to the paragangliomas (**Figures 1E, F**). This may point to a higher radiation dose to the thyroid than the baseline PET scan would suggest. This difference in uptake may be a reflection of slight changes in the biodistribution of the ^68^Ga-chelated versus the ^177^Lu-chelated DOTATATE agent, or due to differing radiopharmaceutical kinetics and organ/tumor washout times since the ^68^Ga and ^177^Lu images were acquired at different times post injection (1 and 24 h, respectively) (15). Furthermore, these findings could also be related to the differences in resolutions of PET imaging for ^68^Ga-DOTATATE compared to planar imaging for ^177^Lu-DOTATATE. These types of discrepancy between Ga-68 and Lu-177 DOTATATE have been previously reported (16).

Other somatostatin analogs such as indium 111 (^111^In) pentetreotide are known to demonstrate physiological uptake into thyroid tissue (17). In addition, increased uptake of ^111^In pentetreotide in a right lateral ectopic thyroid gland located at the carotid bifurcation has also been reported (18). This structure was surgically removed for the concerns of a paraganglioma, but final pathology revealed thyroid tissue with enlarged follicles, although evidence of any autoimmune destruction was not reported. These data may suggest that an increased uptake in the thyroid gland on a diagnostic somatostatin analog imaging study might predict potential thyroid disruption following ^177^Lu-DOTATATE therapy or other forms of PRRT.

^177^Lu-DOTATATE and other radionuclide somatostatin analogs demonstrate high affinity to SSTR-2 (19). SSTR-2 expression has been shown to be substantially prevalent in the thyroid, both in the normal tissue as well as in thyroid tumors (19). In a study by Druckenthaner et al., expression of *SSTR* mRNA was identified in 94% (16 out of 17) of the normal thyroid tissue samples, out of which 82% (14 out of 17) samples expressed *SSTR2* mRNA (19). Somatostatin analogs such as ^68^Ga DOTATOC have also demonstrated uptake in normal thyroid glands (14). Therefore, it is likely that ^177^Lu-DOTATATE may target those thyroid glands that harbor higher levels of SSTR-2.

Apart from pituitary-thyroid axis, endocrine disruption associated with ^177^Lu-DOTATATE therapy has also been noted to affect pituitary-adrenal and pituitary-gonadal axes, albeit most of these effects are transient (9). These data may suggest that the endocrine system may be susceptible to PRRT-induced structural and functional disruption to a variable degree, and the risk of potential disruption might depend on the extent of SSTR-2 expression. In conclusion, ^177^Lu-DOTATATE therapy can be associated with transient or permanent disruption of thyroid function. Identification of uptake patterns in the thyroid gland on pre-treatment diagnostic imaging studies (such as ^68^Ga-DOTATATE) as well as presence of anti-TPO/anti-Tg antibodies may predict the susceptibility of a patient to PRRT-associated induction or exacerbation of thyroid dysfunction. In addition, patients with pre-existing autoimmune thyroid disease might be at a higher risk for accelerated destruction of the SSTR2-positive thyroid tissue following PRRT. Therefore, measurement of TFTs along with thyroid auto-antibody profile (anti-TPO, anti-Tg, and TrAb/TSI) should be considered prior to initiation of PRRT and periodically assessed throughout the course of therapy. Although not performed in our patient, thyroid ultrasonography along with color flow Doppler could serve as a useful tool to assess the physical characteristics as well as vascularity of the thyroid gland among patients with disrupted TFTs. Further studies with prospective data are needed to assess the spectrum, duration, and prognosis of PRRT-associated endocrine disruption, and to evaluate its association with pre-treatment imaging uptake patterns and with endocrine autoimmunity.

## Data Availability Statement

The original contributions presented in the study are included in the article/supplementary material. Further inquiries can be directed to the corresponding author.

## Ethics Statement

The studies involving human participants were reviewed and approved by National Institutes of Health. The patients/participants provided their written informed consent to participate in this study.

## Author Contributions

SG prepared the initial draft of the manuscript and revised the manuscript. MA-J, KP, and JK-G critically reviewed and revised the manuscript and provided input on the endocrine aspects of the manuscript. AJ, MK, JZ, BT, JC, EL, and FL critically reviewed and revised the manuscript and provided input on Lutathera therapy and radiological evaluation. JK-G and FL contributed equally toward the senior authorship of this manuscript. All authors contributed to the article and approved the submitted version.

## Funding

This study was funded by the National Institutes of Health intramural program (Grant number: 1ZIABC011789).

## Conflict of Interest

The authors declare that the research was conducted in the absence of any commercial or financial relationships that could be construed as a potential conflict of interest.
